# Insights from a cross-sectional binational study comparing obesity among nonimmigrant Colombians in their home country and Colombian immigrants in the U.S.

**DOI:** 10.1186/s12889-023-16322-2

**Published:** 2023-08-06

**Authors:** Carlos Devia, Karen R. Flórez, Sergio A. Costa, Terry T-K Huang

**Affiliations:** Center for Systems and Community Design, Graduate School of Public Health and Health Policy, City University of New York, 55 West 125th Street, New York, NY 10027 USA

## Abstract

**Background:**

Latinos in the United States (U.S.) represent a heterogeneous minority population disproportionally impacted by obesity. Colombians in the U.S. are routinely combined with other South Americans in most obesity studies. Moreover, most studies among Latino immigrants in the U.S. solely focus on factors in the destination context, which largely ignores the prevalence of obesity and contextual factors in their country of origin, and warrant transnational investigations.

**Methods:**

Using 2013-17 data from the New York City Community Health Survey (NYC CHS, U.S.) and the National Survey of the Nutritional Situation (ENSIN, Colombia), Colombians that immigrated to the U.S. and are living in NYC (n = 503) were compared to nonimmigrant Colombians living in their home country (n = 98,829). Prevalence ratios (PR) for obesity (BMI ≥ 30 kg/m^2^) by place of residence were estimated using multivariable logistic regression adjusting for socio-demographic characteristics and daily consumption of sugar-sweetened beverages.

**Results:**

The prevalence of obesity was 49% greater for immigrant Colombians living in NYC when compared to nonimmigrant Colombians living in in their home country (PR = 1.49; 95% CI 1.08, 2.07). Colombian immigrant men in NYC were 72% more likely to have obesity compared to nonimmigrant men living in their home country (PR = 1.72; 95% CI 1.03, 2.87). No significant differences were found in the adjusted models among women.

**Conclusions:**

Colombian immigrants in NYC exhibit a higher prevalence of obesity compared to their nonimmigrant counterparts back home and sex strengthens this relationship. More obesity research is needed to understand the immigration experience of Colombians in the U.S. and the underlying mechanisms for sex difference. Public health action focused on women in Colombia and both Colombian men and women immigrants in the U.S. is warranted to avert the long-term consequences of obesity.

## Introduction

The prevalence of obesity in adults continues to increase worldwide, in both high- and low-income countries [1–3]. Between 1975 and 2016, the prevalence of obesity nearly tripled worldwide [2, 3]. In 2016, 1.9 billion adults (18 years and older) were in the overweight category, representing 39% of the world population (39% of men and 40% of women) [3]. In the United States (U.S.), there are disparities in obesity rates by racial and ethnic groups. For example, Latinos, the youngest and fastest growing minority group, have higher age-adjusted rates of obesity compared to non-Latino Whites (46% vs. 41%) [4].

Historically, Latino immigrants to the U.S. generally have lower obesity levels than non-Latino Whites; their U.S.-born counterparts; and other Latinos with greater length of stay in the country [5–7]. However, new research suggests that Latino immigrants may no longer be arriving in the U.S. with healthy weight status because Latin American countries have been undergoing epidemiologic [8, 9] and nutrition transitions [9, 10]. Nonetheless, this remains an understudied domain because despite the diversity in culture and origin from 20 Spanish-speaking countries in Latin America, most obesity research with Latinos in the U.S. have primarily involved Mexican Americans and/or combines data from different Latino groups due to sample size limitations [11–13]. Moreover, most studies among immigrant Latinos compare them to others in the U.S., which largely ignores the prevalence of obesity and contextual factors in the origin context. Given that obesity is a global public health concern and migration may serve as a conduit for this spread, more comparative studies among other Latino sub-groups are warranted to assess how obesity may differ between immigrants and their source populations [6, 14–16].

Colombia has been the largest source of South American immigration to the U.S. for several decades [17]. Colombia is located in the northwest region of South America with a population of over 50 million in 2020, making it the third-most populous country in Latin America only surpassed by Brazil and Mexico [18]. Colombian immigration to the U.S. began in the 1940s and increased substantially in the 1990s due to ongoing domestic armed conflict, encompassing illegal armed groups and drug-related violence, combined with economic recession [17, 19]. In recent years, Colombia has experienced rapid urbanization and integration into global markets [20–22]. This phenomenon is occurring through market policies that include free trade agreements with developed nations or other free investment agreements in the region, which coincide with growing market presence of large food corporations [21, 23]. For example, the annual growth in sales of processed foods and sugar-sweetened beverages (SSBs) in Colombia from 2000 to 2013 was 92.2 kg per capita, representing an increase of 25% [24–26]. These trends have led to diets in Colombia that are increasingly energy-dense but nutrient-poor [27, 28] and are associated with surges in mean body mass index (BMI, kg/m^2^) at the population level [24]. For example, between 2005 and 2015, overweight in the Colombian adult population (18–64 years) increased from 46 to 56% and obesity from 14 to 18% [29].

Approximately 1.2 million Colombian immigrants and their children (first- and second-generation) currently reside in the U.S [30]. The Colombian diaspora in the U.S. is largely concentrated in Florida, New Jersey, and New York City (NYC) [30]. In NYC, socio-demographic characteristics of Colombians are different compared to other large Latino populations (e.g., Puerto Rican, Mexican, Ecuadorian and Dominican) [31, 32]. For example, between 1990 and 2015, the Colombian population had the highest mean age of 41 years, employment rates, and median household income compared to other large Latino groups [31, 32]. Colombians also had significantly lower crude birth rates and are less likely to live in overcrowded households compared to other large Latino groups [31, 32].

Minimal obesity research has been conducted in the Colombian diaspora in the U.S. One study, using data from 2013 to 2017, found that Colombians in NYC had a significantly lower prevalence of obesity when compared to Mexicans and Puerto Ricans residing in NYC [33]. In addition to the paucity of data in general, there are no obesity studies using a transnational perspective [6, 34], comparing the nonimmigrant Colombian population in Colombia with their U.S. counterparts. The present study compares the prevalence of obesity of Colombians immigrants residing in NYC and nonimmigrant Colombians residing in their home country, based on data from two population-based surveys.

## Methods

The current study used publicly available data from two cross-sectional health surveys. The sample of Colombian immigrants in NYC came from the NYC Community Health Survey (NYC CHS) 2013–2017 [35]. Since 2002, the NYC CHS is administered annually by the NYC Department of Health and Mental Hygiene using a random complex telephone-call sample of approximately 10,000 non-institutionalized adults (≥ 18 years), sampled within 34 neighborhood strata in NYC [35]. The survey is administered in English, Spanish, Russian, Cantonese, and Mandarin to selected respondents with landline telephones and mobile phones (added since 2009) [35]. The survey questions are based on the national Behavioral Risk Factor Surveillance System (BRFSS) and provide population-representative estimates of health conditions and risk factors [36].

The sample of nonimmigrant Colombians came from the latest National Survey of the Nutritional Situation in Colombia (ENSIN in Spanish) that was conducted in 2015–2016 by the Colombian Institute of Family Wellbeing (ICBF in Spanish) and the Colombian Ministry of Health [37]. The ENSIN is administered every five years using stratified, probabilistic, multi-stage, and cluster sampling in order to obtain national and sub-regional representativeness (32 states), with oversampling of rural areas and low socio-economic status groups [37]. In ENSIN 2015, a total of 44,202 households were surveyed, based on 4739 groups of 295 strata, representing 99% of the Colombian population [37]. Nutritionists and trained personnel obtained anthropometric measurements from each member of the household and collected information regarding the family’s socio-demographics, dietary behaviors, physical activity, migration history, and food insecurity.

Data from the two population surveys were merged to produce the dataset for analysis. After careful review, several comparable variables were identified for the analysis, including socio-demographic characteristics (i.e., age, sex, education, employment status, and marital status) and one dietary behavior (i.e., consumption of SSBs per day). A secondary analysis was performed using a series of weighted multivariable logistic regression models comparing prevalence ratios (PR) of obesity, adjusted for socio-demographic characteristics and behavior, between adults residing in Colombia and their first-generation counterparts in NYC.

### Institutional review board

The authors declare that all procedures that contributed to this work comply with the ethical standards of the Declaration of Helsinki, revised in 2008. Study methods for the NYC CHS were approved by the NYC Department of Health and Mental Hygiene’s Institutional Review Boards (IRB) and the study methods for the ENSIN were approved by the National Institute of Health of Colombia’s IRB. Participants in both surveys provided informed consent. Data from both surveys are anonymized and accessible to the public by request for secondary analysis. The IRB at the City University of New York deemed this study exempt from review.

### Participants

Adults aged 18 years and over, excluding pregnant women, were the target population of this analysis. Additionally, return migrants were excluded from the analysis to allow a more appropriate comparison between immigrant Colombians and nonimmigrant Colombians. The pooled NYC CHS data from 2013 to 2017 allowed to build representative sample of adult immigrants who self-reported being born in Colombia (n = 503) for comparison with the ENSIN 2015, the latest national data available in Colombia. The ENSIN 2015 included 151,343 individuals, but after excluding minors (n = 49,901), pregnant women (n = 1,403), and individuals who reported living outside Colombia but had returned to live in the country (n = 1,210), the final sample comprised of 98,829 nonimmigrant adults for the entire country, including 13,163 adults living in main Colombian cities with populations of over one million residents (i.e., Bogota, Medellin, Barranquilla and Cali).

### Measures

Obesity was defined as BMI ≥ 30 kg/m^2^ (yes/no) for the analysis. BMI was calculated from self-reported height and weight in the NYC CHS and measured height and weight in the ENSIN. Country of origin was self-reported in NYC CHS (i.e., *Where were you born? Please tell me the country*) and was used to identify Colombian immigrants living in NYC. In addition to the adult population of Colombians residing in their home country, area of residence was used in the ENSIN survey to categorize Colombians that lived in the four main cities in the country (i.e., Bogota, Medellin, Barranquilla and Cali) for urban comparisons with the NYC sample. Age at time of survey was categorized as 18–39, 40–59, and 60 years or over. Sex was self-identified as male or female. Education was classified as less than high school, some college, and college graduate. Employment was categorized as formally employed (i.e., full-time or part-time), self-employed, and unemployed (i.e., this variable excludes individuals who are not in the labor force because they are students, homemakers, retired or unable to work). Marital status was dichotomized as married or living together versus divorced, widowed, separated or never married. Frequency of SSB consumption per day was captured in both surveys and was dichotomized as none or < 1 drink per day versus ≥ 1 drinks per day for the analysis.

### Analysis

This study assessed the relationship between the prevalence of obesity (dependent variable) and place of residence (main explanatory variable) adjusting for obesity risk factors including age, sex, education, employment status, marital status, and daily consumption of SSBs [38]. Additional analysis explored sex differences by place of residence. Descriptive analysis estimated demographic, behavioral, and contextual characteristics of the sample and Pearson’s $x$^2^ tests were used to determine differences between groups. Weights provided by each survey were used to estimate prevalence of obesity to account for potential nonresponse bias and selection probability. The prevalence of obesity was estimated by place of residence (i.e., NYC, the country of Colombia, and main Colombian cities) and by sex. Individual survey weights in the ENSIN were representative of the Colombia population based on the 2005 Colombia National Census [37]. Weights in the NYC CHS were representative of the NYC adult population by sex, race/ethnicity, age, phone type and borough of residence based on the 2015 American Community Survey [36].

Unadjusted and bivariate regressions were used to explore the relationships between obesity and different correlates. The sequence of regression models (parallel models with obesity as the outcome) is as follows. Model 1 compared immigrant Colombians living in NYC versus nonimmigrant Colombians living in their home country. Model 2 examined the relationship between obesity and place of residence, comparing immigrant Colombians living in NYC versus nonimmigrant Colombians living in main Colombian cities only in order to explore differences unique to living in an urban context. Models 3 and 4 identified sex-specific associations between obesity and place of residence. All models adjusted for age, sex, education level, employment status, marital status, and daily SSB consumption.

Analyses were performed using SAS Enterprise Guide (version 7.1) and SAS-callable SUDAAN (version 11.0, Research Triangle Institute, Cary, NC) to adjust for complex samples, taking into account clustering of data, characteristics of the study design, sample weights, and missing data. Results are presented as PR with their respective 95% confidence intervals (95% CI). PR represented the ratio of predicted probabilities calculated from weighted multivariable logistic regression models, using the PREDMARG and PRED_EFF statements in SUDAAN [39]. All statistical tests were two-sided at a significance level of less than 0.05.

## Results

Table 1 provides descriptive statistics for Colombian immigrants living in NYC and nonimmigrant Colombians living in their home country and in main Colombian cities. The eligible participants represented a weighted sample of adult immigrant Colombians residing in NYC (n = 66,648) and nonimmigrant Colombians living their home country (n = 31,886,529) and in main Colombian cities (n = 9,552,846). Based on a Pearson’s $x$^2^ test, Colombians living in NYC were significantly different for all characteristics (all p < 0.001) when compared to Colombians residing in their home country and the four main cities. Results showed that close to half of individuals residing in NYC were between the ages of 40–59 years (44%) and over half of the sample residing in Colombia and in the main cities were between the ages of 18–39 (55% and 54%, respectively). Women represented over half of the sample for Colombians living in NYC (60%), the country of Colombia (51%), and main Colombian cities (52%). Colombian immigrants living in NYC had a higher percentage of people who graduated from college (21%), compared to nonimmigrant Colombians country-wide (9%) and in main Colombian cities (11%). However, a higher percentage of Colombians living in NYC were unemployed (16%) than in Colombia in general (4%) or in main Colombian cities (4%). People married or living with a partner represented over half of the sample among Colombians living in NYC (52%), their home country (55%), and in main Colombian cities (55%). A higher percentage of nonimmigrant Colombians residing throughout the country (30%) and in the main cities (33%) reported drinking one or more servings of SSBs per day, compared to Colombian immigrants living in NYC (24%).

**Table 1:** Participant characteristics^a^

Place of residence	NYC	Colombia	Main Colombian cities^d^		
Sample Size^b^	*n* = 503	*n =* 98,829	*n =* 13,163	P for ✗^2^ test	
Population Size^c^	*n* = 66,684	*n* = 31,886,529	*n* = 9,552,846	P Value^1^	P Value^2^
**Age**				**< 0.001**	**< 0.001**
18–39	27	55	54		
40–59	44	39	39		
60 and over	29	6	6		
**Sex**				**< 0.001**	**< 0.001**
Male	40	49	48		
Female	60	51	52		
**Education**				**< 0.001**	**< 0.001**
Less than high school	31	48	33		
Some college	48	43	56		
College graduate	21	9	11		
**Employment status**				**< 0.001**	**< 0.001**
Employed (Formal)	64	54	71		
Employed (Informal/Self-employed)	20	42	25		
Unemployed	16	4	4		
**Marital status**				**< 0.001**	**< 0.001**
Married or living together	52	55	55		
Divorced, widowed, separated, never married	48	45	45		
**Sugar-sweetened beverages per day**				**< 0.001**	**< 0.001**
≤ 1/day	76	70	67		
> 1/day	24	30	33		

Note. a. The table shows weighted percentages. All weighted proportions reflect New York City population counts from the 2015 American Community Survey and Colombia’s population based on the 2005 Colombia National Census

b. Sample sizes were based on unweighted data

c. Population sizes were based on weighted data

d. Four main cities in Colombia are Bogota, Medellin, Barranquilla, and Cali

^1^ P values for comparisons between Colombian immigrants living in NYC and nonimmigrant Colombians living in their home country

^2^ P values for comparisons between Colombian immigrants living in NYC and nonimmigrant Colombians living in main Colombian cities

Table 2 shows differences across characteristics by sex. There also were significant differences by sex for all characteristics (all p < 0.001) except for marital status. Similar to the patterns above, among both men and women, Colombians immigrants living in NYC were older, more educated, more likely to be unemployed, and less likely to consume SSBs.

**Table 2:** Participant characteristics by sex^a^

Sex	Females					Males				
Place of Residence	NYC	Colombia	Main Colombian cities^d^			NYC	Colombia	Main Colombian cities^d^		
Sample Size^b^	n = 311	n = 50,403	n = 7229	P for ✗^2^ test		n = 192	n = 48,426	n = 6193	P for ✗^2^ test	
**Population Size** ^**c**^	**n = 40,329**	**n = 17,619,379**	**n = 5,306,884**	**P Value** ^**1**^	**P Value** ^**2**^	**n = 26,356**	**n = 14,607,044**	**n = 4,364,880**	**P Value** ^**1**^	**P Value** ^**2**^
**Age**				**< 0.001**	**< 0.001**				**< 0.001**	**< 0.001**
18–39	28	56	55			26	58	58		
40–59	39	38	38			52	36	36		
60 and over	33	6	7			22	6	6		
**Education**				**< 0.001**	**< 0.001**				**< 0.001**	**< 0.001**
Less than high school	36	46	32			24	48	33		
Some college	47	44	56			50	43	56		
College graduate	17	10	12			26	9	11		
**Employment status**				**< 0.001**	**< 0.001**				**< 0.001**	**< 0.001**
Employed (Formal)	66	61	72			62	50	69		
Employed (Informal/Self-employed)	17	35	23			23	45	26		
Unemployed	17	4	4			15	5	5		
**Marital status**				0.38	0.003				0.04	0.38
Married or living together	50	53	52			55	59	58		
Divorced, widowed, separated, never married	50	47	48			45	41	42		
**Sugar-sweetened beverages per day**				**< 0.001**	**< 0.001**				**0.001**	**< 0.001**
≤ 1/day	81	75	74			68	65	61		
> 1/day	19	25	26			32	35	39		

Note. a. The table shows weighted percentages. All weighted proportions reflect New York City population counts from the 2015 American Community Survey and Colombia’s population based on the 2005 Colombia National Census

b. Sample sizes were based on unweighted data

c. Population sizes were based on weighted data

d. Four main cities in Colombia are Bogota, Medellin, Barranquilla, and Cali

^1^ P values for comparisons between Colombian immigrants living in NYC and nonimmigrant Colombians living in their home country

^2^ P values for comparisons between Colombian immigrants living in NYC and nonimmigrant Colombians living in main Colombian cities

### Obesity prevalence

**Fig. 1:**
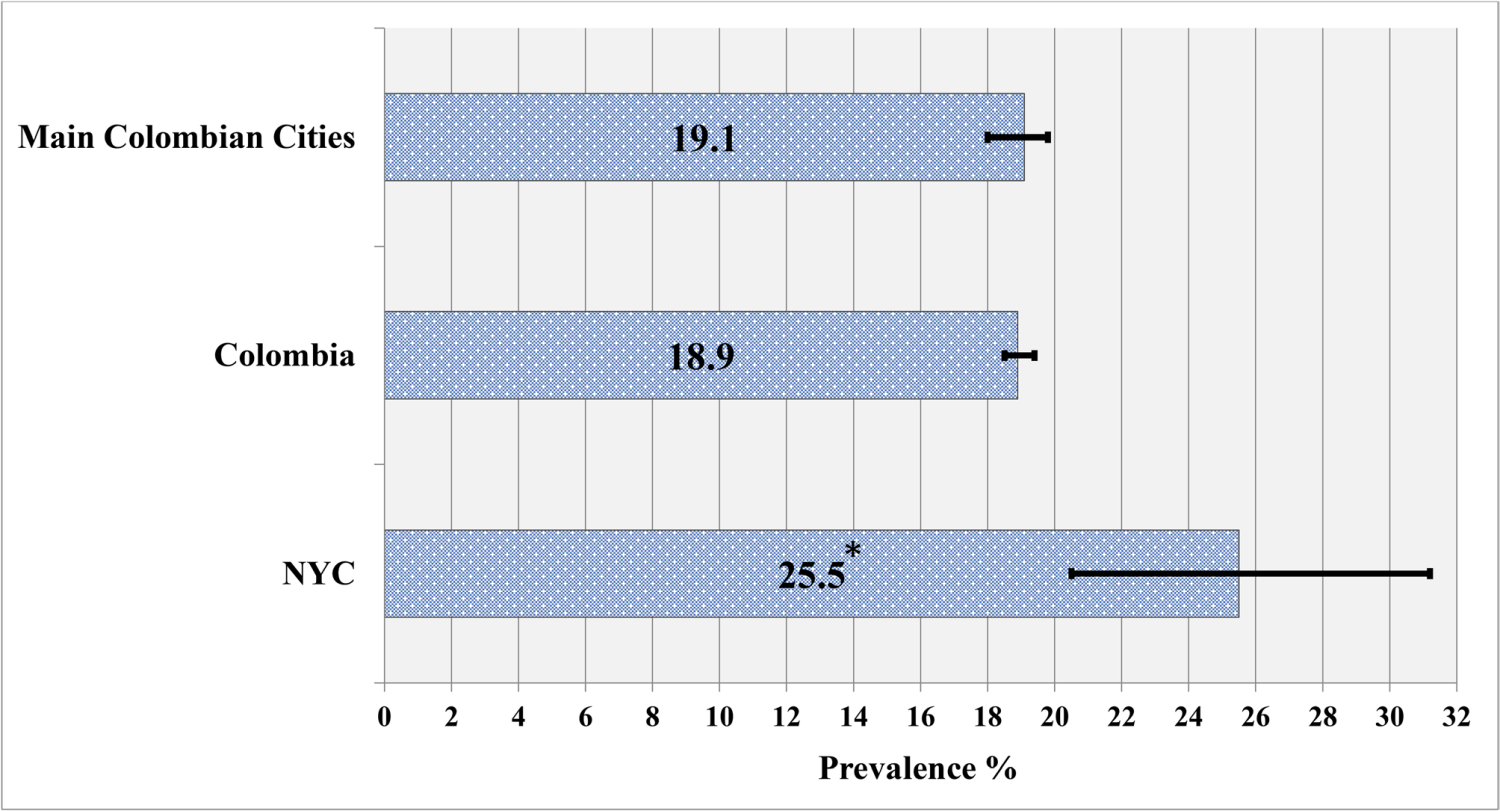
Prevalence of obesity (BMI ≥ 30 kg/m^2^) by place of residence Notes. * Immigrant Colombians living in NYC had a significantly higher prevalence of obesity compared to nonimmigrant Colombians living in their home country (p < 0.01) or in the four main Colombian cities (p = 0.01)

**Fig. 2:**
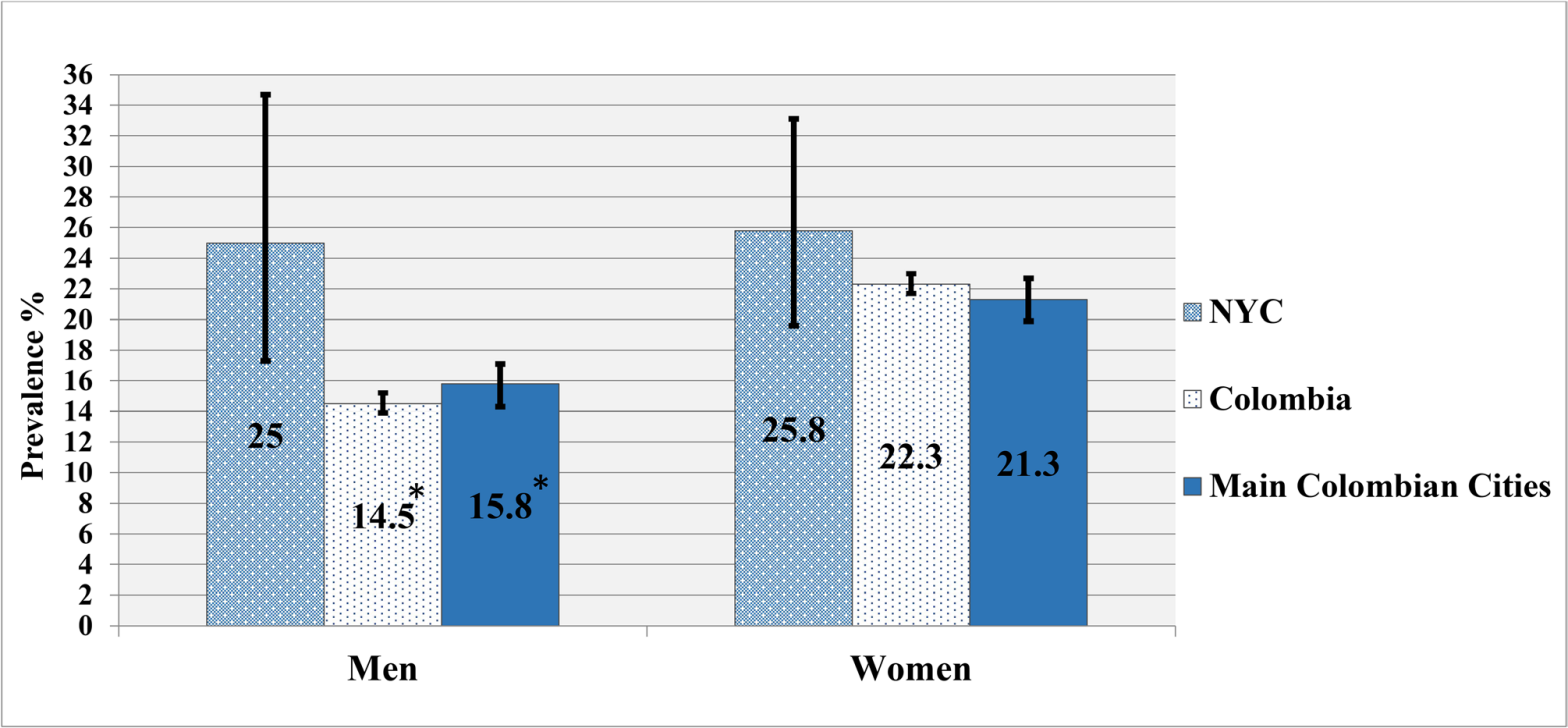
Prevalence of obesity (BMI ≥ 30 kg/m^2^) by sex and place of residence Notes. * Immigrant Colombian men living in NYC had a significantly higher prevalence of obesity compared to nonimmigrant Colombian men living in Colombia (p = 0.02) or in the four main Colombian cities (p < 0.05). No such significant differences were found among women

Figures 1 and 2 show the weighted prevalence of obesity in the total sample and within sex groups by place of residence. The prevalence of obesity among Colombian immigrants living in NYC (25.5%; 95% CI 20.5, 31.2) was significantly higher compared to nonimmigrant Colombians living in their home country (18.9%; 95% CI 18.5, 19.4) and in the main cities (19.1%; 95% CI 18.0, 19.8). There were also differences by sex for some groups. Colombian immigrant men living in NYC (25.0%; 95% CI 17.3, 34.7) had a significantly higher prevalence of obesity compared to nonimmigrant Colombian men country-wide (14.5%; 95% CI 13.9, 15.2) and in the main cities (15.8%; 95% CI 14.2, 16.8). However, there were no significant differences among Colombian immigrant women living in NYC (25.8%; 95% CI 19.6, 33.1) compared to nonimmigrant Colombian women living across Colombia (22.7%; 95% CI 22.0, 23.4) or in the main cities (21.7%; 95% CI 20.3, 23.2).

### Regression models of obesity and place of residence

Table 3 shows the association of obesity with place of residence with and without adjustment for socio-demographic characteristics and daily SSBs consumption. The unadjusted model showed that the prevalence of obesity was 36–37% greater for immigrant Colombians living in NYC when compared to nonimmigrant Colombians country-wide (PR = 1.37; 95% CI 1.13, 1.70) or main Colombian cities (PR = 1.36; 95% CI 1.09, 1.69). After adjusting for socio-demographic and dietary correlates (Models 1 and 2), the prevalence of obesity was 49–65% greater for immigrant Colombians living in NYC when compared to nonimmigrant Colombians living in in their home country (PR = 1.49; 95% CI 1.08, 2.06) and in main Colombian cities (PR = 1.65; 95% CI 1.03, 2.64). Models 3 and 4 compared Colombians living in NYC with their acounterparts in Colombia among men vs. women, respectively. The association of obesity with place of residence among Colombian men was like that of the overall sample, where those living in NYC had a 71% higher rate of obesity than those living in Colombia country-wide (PR = 1.71; 95% CI 1.03, 2.86). However, no significant difference was found among women (PR = 1.27; 95% CI 0.85, 1.88). Similar results were found for sex comparisons between Colombians immigrants in NYC and nonimmigrant Colombians living in the main Colombian cities (data not shown).

**Table 3:** Crude and adjusted prevalence ratios (PR) for obesity by location of residence

	Unadjusted obesity	Unadjusted obesity	Model 1	Model 2	Model 3	Model 4
Model N	*n* = 15,765	*n* = 11,446	*n* = 4038	*n* = 853	*n* = 2524	*n* = 1514
	PR (95% CI)	PR (95% CI)	PR (95% CI)	PR (95% CI)	PR (95% CI)	PR (95% CI)
**Place of residence**						
Colombia	Referent		Referent		Referent	Referent
NYC	1.37 (1.13, 1.70)**		1.49 (1.08, 2.06)**		1.71 (1.03, 2.86)*	1.27 (0.85, 1.88)
**Place of residence**						
Main Colombian cities^a^		Referent		Referent		
NYC		1.36 (1.09, 1.69)**		1.65 (1.03, 2.64)*		
**Gender**						
Male			Referent	Referent		
Female			1.44 (1.12, 1.85)***	1.14 (0.73, 1.78)		
**Age**						
18–39			Referent	Referent	Referent	Referent
40–59			1.22 (0.94, 1.57)	1.04 (0.63, 1.72)	1.29 (0.88, 1.88)	1.11 (0.78, 1.57)
60 and over			1.12 (0.70, 1.79)	1.11 (0.58, 2.13)	0.81 (0.41, 1.63)	1.36 (0.72, 2.59)
**Education**						
Less than high school			Referent	Referent	Referent	Referent
Some college			0.92 (0.71, 1.19)	1.05 (0.66, 1.67)	0.97 (0.68, 1.38)	0.85 (0.60, 1.21)
College graduate			0.58 (0.38, 0.87)**	0.41 (0.22, 0.75)***	0.75 (0.42, 1.33)	0.48 (0.27, 0.85)**
**Employment**						
Employed (Formal)			Referent	Referent	Referent	Referent
Employed (Informal/Self-employed)			0.84 (0.66, 1.08)	0.61 (0.37, 1.02)	0.79 (0.56, 1.10)	0.94 (0.67, 1.34)
Unemployed			1.28 (0.87, 1.88)	1.74 (1.09, 2.79)*	1.35 (0.79, 2.30)	1.24 (0.73, 2.13)
**Marital Status**						
Divorced, widowed, separated, never married			Referent	Referent	Referent	Referent
Married or living together			1.13 (0.88, 1.46)	0.89 (0.55, 1.45)	1.27 (0.85, 1.91)	1.02 (0.73, 1.44)
**Sugar-sweetened beverages per day**						
≤ 1/day			Referent	Referent	Referent	Referent
> 1/day			1.03 (0.82, 1.30)	0.80 (0.52, 1.25)	1.05 (0.77, 1.44)	1.00 (0.71, 1.41)

***p < 0.001; **p < 0.01; *p < 0.05

a. Four main cities in Colombia are Bogota, Medellin, Barranquilla, and Cali

Model 1. Colombians immigrants in NYC vs. nonimmigrant Colombians in their home country.

Model 2. Colombians immigrants in NYC vs. nonimmigrant Colombians in main Colombian cities

Model 3. Colombian immigrant men in NYC vs. nonimmigrant Colombian men in their home country

Model 4. Colombian immigrant women in NYC vs. nonimmigrant Colombian women in their home country

Obesity is defined as BMI ≥ 30 kg/m^2^

Adjusted for age, sex, education level, employment status, marital status, and SSBs consumption

## Discussion

This is one of the first binational comparative study of obesity among Colombians who immigrated to NYC and nonimmigrant Colombians in their home country. This study found that Colombian immigrants residing in NYC had a higher BMI and prevalence of obesity than nonimmigrant Colombians in Colombia. Notably, this difference was primarily driven by the different rates of obesity among men based on place of residence.

Indeed, this study showed that the prevalence of obesity was already higher among women than men residing in Colombia overall (22% vs. 14%) or in the four main Colombian cities (21% vs. 15%), suggesting that the nutrition and epidemiologic transition in Colombia may have impacted Colombian women to a greater extent. Similar differences by sex have been reported in other studies in Colombia and other Latin American countries [40, 41]. However, of note, the prevalence of obesity among Colombian women and men living in NYC was similar (26% vs. 25%, respectively). More research is needed to elucidate how the process of immigration may differently affect Colombians. The current study suggest that education level and employment status are potential pathways for weight status among Colombian immigrants [33]. These factors should be researched further in terms of how they relate to new social norms and lifestyle in the U.S., migration networks, and disease status or disabilities. In addition, little intervention research has focused on Latino men living in the U.S. This study shows the imperative to pay closer attention to the high rates of obesity in this population.

The fact that nonimmigrant Colombian women living their home country have a similar prevalence of obesity as Colombian immigrant women living in NYC is alarming, given that population obesity rates are much higher in the U.S. in general. Previous studies using primary data analysis and other research methods have identified factors that may explain the higher prevalence of obesity among Colombian women, including preferences in body size unique to women in Latino cultures [42, 43], hormonal differences [41], excess gestational weight gain or post-partum weight retention [41], and wealth inequities [44]. Similar to other Latin American countries, Colombia has adopted national strategies to combat obesity, including an obesity prevention policy reform (Law No 1355, Colombia 2009), restrictions on fat (trans fatty acids, saturated fats) in foods, the taxation of processed food and SSBs, promotion of physical activity in open spaces through bike paths, and front-of package labeling [45, 46]. However, there is still no specific intervention aimed at Colombian women at the national level. It is critical to continue researching this phenomenon, as Colombian women need better public health interventions to prevent and address obesity in both Colombia and the U.S.

Prior research has shown that the prevalence of obesity among Colombians living in NYC is higher compared to non-Latino people living in NYC (25% vs. 21%) [47]. There are several reasons why this may be the case. Multiple studies based on primary analysis and other research methods suggest that the risk for overweight and obesity increases as first-generation Latinos are exposed to obesogenic environments [48] and acculturate to mainstream lifestyle behaviors in the U.S. (e.g., high energy diets without compensating for adequate levels of physical activity) [7, 49–53]. The tendency for recently arrived Latinos to have lower-than-average rates of obesity and some illnesses despite living in disadvantaged conditions is part of what has been termed the Hispanic Paradox [52]. Most notable for mortality risk, this paradox suggests that better lifestyle habits of immigrants result in better health outcomes and that this advantage holds regardless of gender and age [52–54]. However, this relative advantage wanes with age of arrival, longer duration of residence in the U.S., and by the second generation [7, 50, 52, 53]. The Hispanic Paradox may, in part, explain the high rate of obesity among Colombian men in NYC but does not explain the lack of differences in obesity rates by place of residence among Colombian women. In fact, some studies suggest that the benefits of the Hispanic Paradox seem to be disappearing for newly arriving immigrants from some countries in Latin America experiencing high rates of obesity (e.g., Mexico and Chile) [5, 55]. More research is required to understand if this may be occurring among Colombian women.

Finally, the results of this study do not show major differences based on the urban context, as comparisons of the NYC sample with either the full Colombian sample or the sample limited to the four main Colombian cities did not reveal any differences. This suggests that the nutrition and epidemiologic transition in Colombia may have become widespread in recent years, consistent with the economic growth and globalization seen in the country. Work in other Latin American countries document higher count of all types of food stores in urban areas; however, the most drastic changes have been observed in non-urban areas and socioeconomically deprived areas [56], though the proliferation of new convenience stores that typically have more food items that are calorie dense but nutrient poor [57]. A more detailed analysis of these shifts across Colombia seems warranted.

In addition to being the first study of its kind, a major strength of this paper lies in the fact that the binational data came from population-representative samples of Colombia (ENSIN 2015) and NYC (NYC CHS 2013–2017) allowing comparisons from both sending and receiving countries. Each survey used rigorous and universally accepted complex sampling methods, allowing for the unique opportunity to confer sufficient power for comparative analysis. Second, this study analyzed data from immigrant Colombians and their compatriots who do not immigrate. This approach allowed a more appropriate comparison group for outcome, behavior, and socio-demographics in order to assess if migration to the U.S. is associated with obesity. Third, although not the focus of this paper, we note that the direction and strength of the associations between obesity and socio-demographic variables were similar to those reported in previous studies in Colombia and the U.S., which confer external validity to the findings. Finally, this study used six potentially confounding variables common to both surveys for risk adjustment, including socio-demographics variables and one diet-related variable.

This study also has some weaknesses. First, this study used cross-sectional data; therefore, it is not possible to establish causality. Second, unlike the ENSIN dataset, height and weight was self-reported in the NYC CHS dataset, which might have introduced measurement error due to recall and social desirability biases [58]. However, studies suggest that self-reported BMI is typically underreported, but Latino immigrants in the U.S. are less likely to underreport BMI compared to non-Latino Whites [59]. Another limitation is the lack of migration information available in both datasets. For example, it was not possible to assess length of stay in the U.S. for immigrant Colombians residing in NYC. On the other hand, it was not possible to assess migration networks (i.e., relatives that lives in NYC or abroad) that could influence lifestyle behaviors of nonimmigrant Colombians. Furthermore, there may still be residual or other unmeasured confounders not captured in this study (e.g., acculturation measures, other health behaviors, and medical diagnoses). Finally, the findings of this study are specific to NYC and may be not generalizable to other Colombian immigrants in the U.S.

## Conclusion

Despite the limitations, the study findings are a unique contribution to transnational obesity research, particularly for a Latino population in the U.S. that is understudied. This study contributes to further understanding of the heterogeneity of obesity and related risks for Latino immigrants in the U.S. Colombians in NYC represent one of the largest South American populations in the U.S., and this study shows that they are experiencing disproportionately high rates of obesity and that any protection among men in Colombia is no longer present after immigration to the U.S. Future research is needed to understand the experiences of Colombians in the U.S., including acculturation, racial discrimination and structural barriers (e.g., policies that restrict public health and healthcare services). While Colombians living in NYC experience higher rates of obesity compared to Colombians in their home country, this comparison also unmasked disparities by sex, with significant differences by place of residence found only for men but not for women. This warrants further research into the biological, behavioral, environmental, and socio-cultural mechanisms underlying such sex disparity. Collectively, the findings of this study support the urgent need for public health research and action strongly focused on adult women in Colombia and both Colombian men and women in the U.S. to avert the long-term consequences of obesity.

## Data Availability

The databases that allowed this analysis are available for public access and can be obtained by request. The New York City Department of Health and Mental Hygiene, Community Health Survey (NYC CHS), years 2013–2017 is available at https://www.nyc.gov/site/doh/data/data-sets/community-health-survey.page The Colombian Institute of Family Wellbeing, National Survey of the Nutritional Situation in Colombia (ENSIN), year 2015 is available at https://www.icbf.gov.co/bienestar/nutricion/encuesta-nacional-situacion-nutricional The data generated and analyzed for the current study are available at reasonable request to the corresponding author.
